# Lower limb compression in acute heart failure and predominant tissue congestion: rationale and design of the COMPRESSION-HF trial

**DOI:** 10.1093/eschf/xvag214

**Published:** 2026-08-12

**Authors:** José Manuel Civera, Elena Chover, Patricia Castro, José Pérez Silvestre, David García Escrivá, Pau Llàcer, Esteban Pérez Pisón, Marta Cobo Marcos, Juan Carlos López Azor, Francisco Pastor Pérez, Amparo Martínez López, Clara Simón Ramón, Alba Villalobos Abelló, Julio Núñez, Rafael de la Espriella

**Affiliations:** Cardiology Department, Hospital Clínico Universitario de Valencia, INCLIVA, Avda. Blasco Ibáñez 17, Valencia 46010, Spain; Internal Medicine Department, Hospital General Universitario de Valencia, Valencia, Spain; Nursing Department, Universitat de València, Valencia, Spain; Cardiology Department, Hospital Clínico Universitario de Valencia, INCLIVA, Avda. Blasco Ibáñez 17, Valencia 46010, Spain; Internal Medicine Department, Hospital General Universitario de Valencia, Valencia, Spain; Internal Medicine Department, Hospital General Universitario de Valencia, Valencia, Spain; Internal Medicine Department, Hospital Universitario Ramón y Cajal, Madrid, Spain; Internal Medicine Department, Hospital Universitario Ramón y Cajal, Madrid, Spain; Cardiology Department, Hospital Universitario Puerta de Hierro Majadahonda, Majadahonda, Madrid, Spain; Cardiology Department, Hospital Universitario Puerta de Hierro Majadahonda, Majadahonda, Madrid, Spain; Cardiology Department, Hospital Clínico Universitario Virgen de la Arrixaca, Murcia, Spain; Cardiology Department, Hospital Clínico Universitario Virgen de la Arrixaca, Murcia, Spain; Cardiology Department, Hospital de la Santa Creu i Sant Pau, Barcelona, Spain; Cardiology Department, Hospital de la Santa Creu i Sant Pau, Barcelona, Spain; Cardiology Department, Hospital Clínico Universitario de Valencia, INCLIVA, Avda. Blasco Ibáñez 17, Valencia 46010, Spain; Department of Medicine, Universitat de València, Avenida Blasco Ibáñez 15, 46010 Valencia, Spain; CIBER Cardiovascular, Avenida Monforte de Lemos 3-5, 28029 Madrid, Spain; Cardiology Department, Hospital Clínico Universitario de Valencia, INCLIVA, Avda. Blasco Ibáñez 17, Valencia 46010, Spain; CIBER Cardiovascular, Avenida Monforte de Lemos 3-5, 28029 Madrid, Spain

**Keywords:** Acute heart failure, Congestion, Oedema, Natriuresis, Compression therapy, Interstitial fluid

## Abstract

**Aims:**

Acute decompensated heart failure (ADHF) is often treated with diuretic agents in both the inpatient and ambulatory settings, yet fluid overload frequently persists and is associated with rehospitalization and death. In patients with predominant interstitial fluid expansion but without intravascular overload, increasing the loop diuretic dose or adding a second diuretic may cause intravascular volume depletion, electrolyte disturbance, and worsening renal function without clearing the excess tissue fluid. COMPRESSION-HF tests whether adding lower limb compression to standard diuretic treatment improves early decongestion in patients with ADHF, predominant peripheral oedema, and inferior vena cava (IVC) diameter ≤21 mm.

**Methods:**

COMPRESSION-HF (NCT06418932) is an investigator-initiated, multicentre, randomized, double-blind, sham-controlled trial in which adults with ADHF, bilateral leg oedema (grade II or higher of IV), N-terminal pro–B-type natriuretic peptide (NT-proBNP >1000 pg/ml), and IVC diameter ≤21 mm were assigned 1:1 to active bilateral lower limb compression (two-layer inelastic–elastic bandaging; manufacturer-specified nominal ankle pressure, approximately 20 mm Hg) plus parenteral furosemide or to sham bandaging plus parenteral furosemide, for up to 72 h. The two co-primary endpoints are 24-h urinary sodium excretion and change in body weight from baseline. Secondary endpoints include changes in lower limb circumference, clinical congestion score, and IVC diameter, together with NT-proBNP at 72 h, cumulative 72-h furosemide-equivalent dose, carbohydrate antigen 125 (CA-125) at day 15 ± 3, and 30-day safety. Continuous endpoints will be analysed with mixed-effects analysis of covariance or repeated-measures models including centre as a random intercept. Recruitment is complete (106 patients randomized); the trial was powered to detect a 20 mmol between-group difference in 24-h urinary sodium excretion.

**Conclusions:**

COMPRESSION-HF will show whether adding lower limb compression to standard loop diuretic treatment increases natriuresis and weight loss in patients with ADHF and predominant peripheral oedema, and will describe the relationship between tissue congestion, intravascular refilling, and the diuretic response.

## Introduction

Congestion is the main reason why patients with acute decompensated heart failure (ADHF) seek urgent medical care, and persistent congestion is strongly associated with death and readmission.^1–3^ However, congestion is not a uniform state. Excess extracellular fluid is distributed between two compartments that remain in dynamic equilibrium: the intravascular compartment, represented by plasma volume and venous pressures, and the extravascular compartment, represented by interstitial and third-space fluid. Although many patients have both intravascular and tissue congestion, the relative contribution of each compartment varies and influences the response to decongestive treatment.^4,5^

Loop diuretics promote renal sodium and water excretion and initially reduce plasma volume. When the response is sustained, vascular filling pressures fall, Starling forces at the microcirculation are modified, and interstitial fluid is expected to return to the vascular compartment for subsequent renal elimination.^6^ Decongestion can therefore be understood as a sequential process in which the intravascular compartment is depleted first and the interstitial compartment second. In clinical practice, this sequence is often incomplete. Plasma refill decreases during decongestion, and the interstitium is not a passive reservoir of free water. Its glycosaminoglycan-rich matrix binds sodium and water and, when oedema is established, becomes more compliant, favouring persistent tissue fluid accumulation.^7,8^

In patients with predominant tissue congestion and a relatively non-congested intravascular compartment, further increases in loop diuretic dose or the addition of a second diuretic may produce intravascular volume depletion, electrolyte disturbances, and worsening renal function without achieving proportional tissue decongestion.^9^ Pharmacological strategies intended to improve fluid transfer or restore effective circulating volume, including hypertonic saline, albumin, and osmotic approaches, have yielded inconsistent results and lack robust randomized support.^10–15^ Direct strategies to facilitate interstitial fluid mobilization therefore remain an unmet clinical need.^6,7^

Lower limb compression offers a simple, non-pharmacological strategy to facilitate tissue decongestion. By increasing interstitial hydrostatic pressure and venous return, compression may promote movement of interstitial fluid into the vascular and lymphatic compartments, increasing the substrate available for renal excretion during loop diuretic therapy.^16^ This rationale was tested in a single-centre pilot study of 20 patients with ADHF. The median baseline inferior vena cava (IVC) diameter was 14.5 mm [interquartile range (IQR), 14–20] in patients with an IVC ≤21 mm and 27.0 mm (IQR, 25–28) in those with an IVC >21 mm. Following compression, IVC diameter increased by 2.4 mm [95% confidence interval (CI), 1.0–3.8; *P* < .001] in the lower-IVC subgroup and by 0.8 mm (95% CI, −0.6 to 2.2; *P* = .611) in the higher-IVC subgroup (*P* for interaction <.001). This pattern suggested greater capacity for intravascular refill in patients without marked baseline central venous distension, and the refill response was accompanied by greater natriuresis and clinical decongestion.^17^

These findings generated the hypothesis of COMPRESSION-HF. The trial was designed to test whether adding lower limb compression to standard parenteral loop diuretic therapy improves early decongestion in patients with decompensated heart failure, predominant tissue congestion, and no marked intravascular congestion, defined by bilateral tibio-malleolar oedema and IVC diameter ≤21 mm. Here, we present the rationale and design of the trial.

## Methods

### Study design and oversight

**Figure 1:**
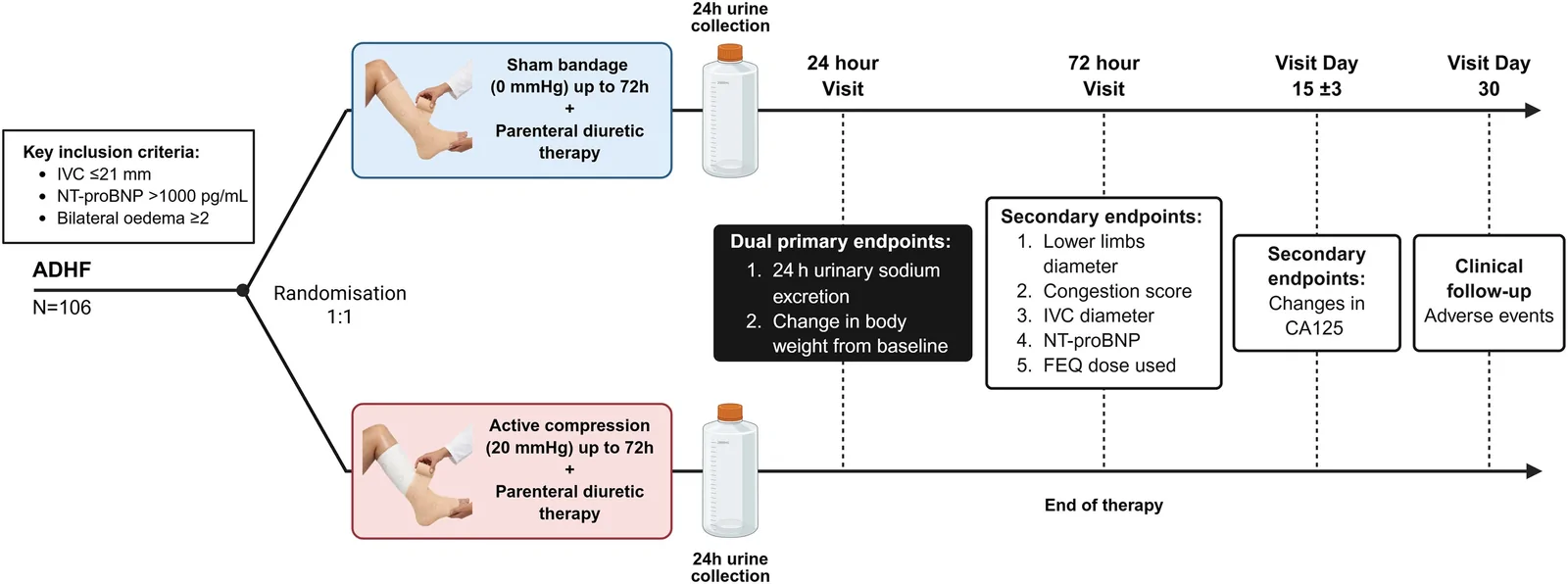
Overall study design and schedule of assessments from screening to 30-day follow-up

COMPRESSION-HF is an investigator-initiated, multicentre, randomized, double-blind, sham-controlled trial to assess whether adding bilateral lower limb compression plus parenteral diuretic therapy, compared with sham compression plus parenteral diuretic therapy, improves early decongestion in patients with ADHF and predominant tissue congestion (*Figure 1*). Patients are randomized 1:1, and the intervention is applied for a minimum of 24 h and a maximum of 72 h, with follow-up to 30 days. Structured assessments of clinical congestion, body weight, lower limb circumference, urinary sodium excretion, diuretic exposure, renal function, NT-proBNP, carbohydrate antigen 125 (CA-125), IVC diameter, clinical congestion score, and safety are performed at scheduled visits.

Ethical approval was obtained from the relevant ethics committee and the Spanish competent authority. The study is conducted in adherence to the principles of the Declaration of Helsinki, Good Clinical Practice guidelines, Regulation (EU) 2017/745 on medical devices, and applicable Spanish regulatory requirements. Written informed consent is obtained from all participants before any study-specific procedure. The trial is registered at ClinicalTrials.gov (NCT06418932). Enrolment commenced on 27 May 2024 and was concluded on 21 April 2026, with 106 patients randomized.

### Study population

Eligible patients are adults with ADHF requiring parenteral loop diuretic therapy and a congestion phenotype characterized by bilateral lower limb oedema without marked intravascular congestion. Patients can be enrolled from inpatient or ambulatory settings when all eligibility criteria are fulfilled.

The key selection criterion is the coexistence of clinically relevant peripheral tissue congestion and an IVC diameter ≤21 mm. This threshold was chosen to enrich the trial for patients in whom tissue-to-vascular fluid recruitment, rather than additional vascular unloading alone, is the expected limiting step for decongestion. Screening also requires NT-proBNP >1000 pg/ml and excludes conditions in which compression is unsafe or that may confound interpretation of decongestion (*Table 1*).

**Table 1:** Key eligibility criteria

Inclusion criteria	Exclusion criteria
Age ≥18 years	Admission to an intensive care unit
Clinical diagnosis of acute decompensated heart failure within 96 h after initiation of parenteral diuretic therapy	Renal transplant, chronic kidney disease Stage 5, estimated glomerular filtration rate <15 ml/min/1.73 m^2^, dialysis, or need for ultrafiltration
Treatment with furosemide ≥40 mg within the preceding 24 h	Absent peripheral pulses
NT-proBNP >1000 pg/ml at any time since onset of decompensation	Ankle–brachial index <0.9
Bilateral tibio-malleolar oedema grade II or higher of IV at inclusion	History of severe peripheral arterial disease
Inferior vena cava diameter ≤21 mm on subcostal echocardiography at screening	Previous intolerance to compressive bandaging
	Heart failure secondary to acute myocardial infarction

NT-proBNP, N-terminal pro–B-type natriuretic peptide.

Eligibility requires a clinical diagnosis of ADHF within 96 h after initiation of parenteral diuretic therapy and treatment with at least 40 mg of furosemide during the preceding 24 h. The 96-h criterion therefore defines the maximum interval from treatment initiation to enrolment rather than a minimum duration of previous therapy.

### Randomization and blinding

After consent and confirmation of eligibility, patients are randomized 1:1 through an electronic platform (Pinvestiga) using permuted blocks. Patients, treating clinicians responsible for clinical decisions, outcome assessors, and investigators remain blinded to treatment allocation. Active and sham systems have a similar external appearance, and personnel responsible for bandage application are separated from subsequent clinical, echocardiographic, urinary, biomarker, and safety assessments.

### Standard care

All patients receive parenteral loop diuretic therapy and background heart failure care according to the treating physician’s judgement and contemporary guideline-based practice. Furosemide dose, route, timing, escalation, and transition to oral diuretics are not protocol-fixed. Diuretic exposure is prospectively recorded and converted to furosemide equivalents when required.

### Active compression intervention

**Figure 2:**
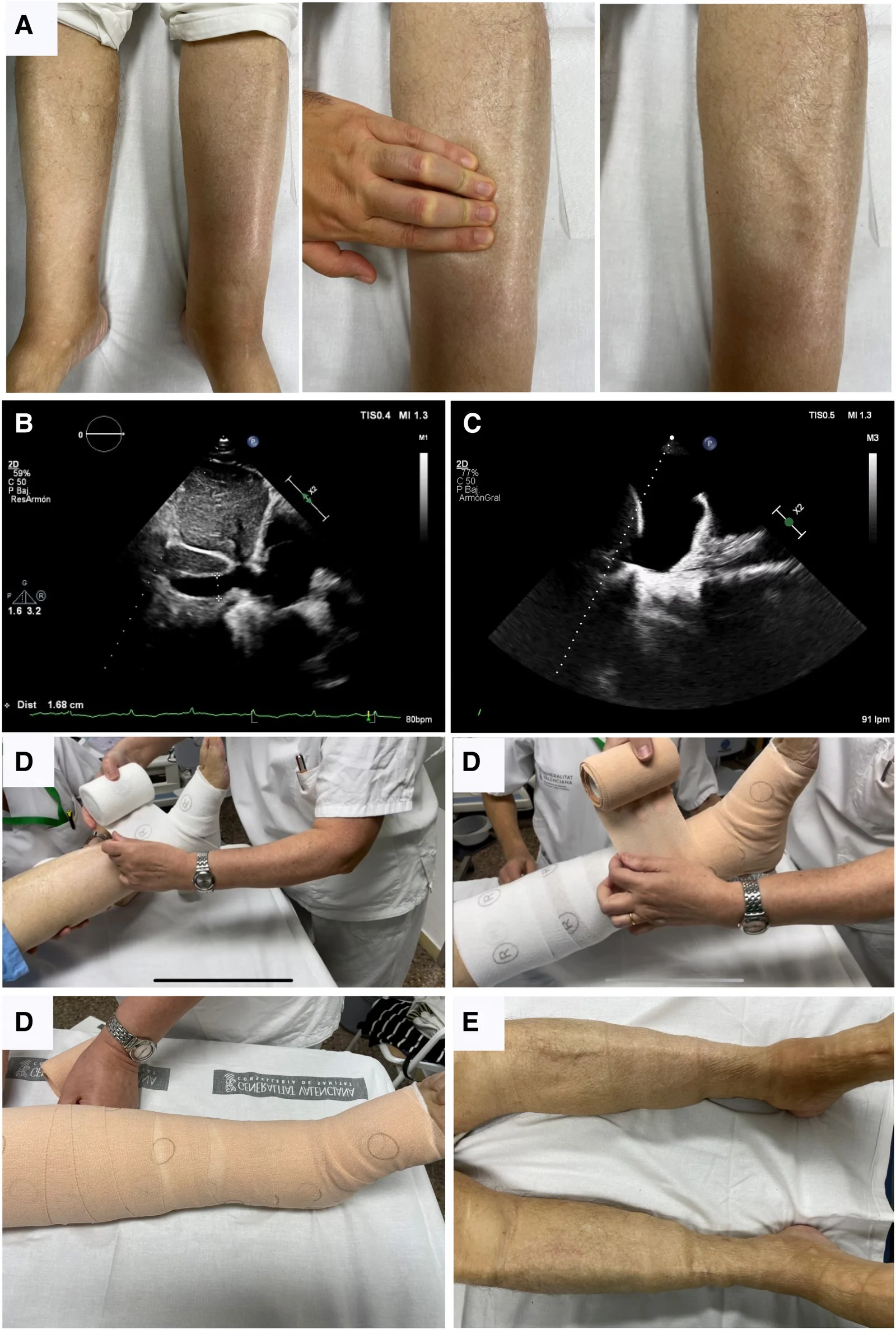
Illustrative case of a patient with predominant tissue congestion treated with lower limb compression in the COMPRESSION-HF trial. A 79-year-old man presented with progressive dyspnoea and lower limb oedema. Baseline serum creatinine was 2.3 mg/dl (estimated glomerular filtration rate 28 ml/min/1.73 m^2^), N-terminal pro–B-type natriuretic peptide was 2635 pg/ml, and carbohydrate antigen 125 was 87 U/ml. (*A*) Bilateral pitting oedema of the lower limbs at presentation; the central panel shows the pitting sign after digital pressure. (*B*) Subcostal echocardiography showing a non-dilated inferior vena cava (17 mm), consistent with the absence of intravascular congestion. (*C*) Lung ultrasound demonstrating pleural effusion. The patient met all inclusion criteria and no exclusion criteria and was randomized in COMPRESSION-HF, with allocation to the active compression arm. (*D*) Application of the bilateral two-layer (inelastic–elastic) lower limb compression bandage by trained staff. (*E*) After 48 h of treatment, marked clinical improvement with complete resolution of the lower limb oedema

Patients allocated to the active intervention receive bilateral lower limb compression with UrgoK2 Lite (Urgo Medical), a commercially available two-layer multicomponent bandage system designed to provide a manufacturer-specified nominal ankle pressure of approximately 20 mm Hg when applied according to the instructions for use. Interface pressure is not measured instrumentally in individual participants. The system includes a padded short-stretch inelastic layer and a cohesive long-stretch elastic layer. The short-stretch component provides stiffness during movement, whereas the cohesive outer layer maintains resting pressure and secures the bandage. Integrated PresSure System visual indicators guide the intended stretch and overlap during application (*Figure 2*).

Bandages are applied bilaterally by trained study personnel according to the manufacturer’s instructions (*Figure 2*). Kit size is selected according to ankle circumference measured approximately 2 cm above the malleolus, and the foot is positioned at 90°. The first layer is anchored at the base of the toes, applied with a figure-of-eight technique around the heel, and wrapped spirally from the ankle to just below the knee. The second cohesive layer is applied over the first using the same technique. Application fidelity is checked during initial placement and at each 24-h renewal by stretching the oval PresSure System indicators until they become circular and by following the printed overlap guides. The bandage is renewed every 24 h and maintained for 24–72 h unless discontinued earlier for intolerance, safety concerns, patient withdrawal, clinical indication, or completion of the intervention period.

### Sham compression

Patients allocated to the control group receive visually matched sham bandaging without therapeutic compression. The sham system consists of a non-compressive padded dressing covered by elastic tubular mesh, designed to reproduce the external appearance of active compression without delivering clinically relevant pressure. Sham bandaging is applied bilaterally and renewed on the same schedule as active compression.

Lower limb circumference is measured bilaterally at prespecified points 5 and 10 cm above the ankle at baseline, before initial bandage application, and at 24 and 72 h. At each follow-up assessment, the active or sham bandage is removed, and circumference is measured before application of the replacement bandage or final discontinuation of the intervention.

### Endpoints

The trial has two co-primary endpoints, both assessed at 24 h after randomization:

1. Change in body weight from baseline to 24 h.
2. Total urinary sodium excretion during the first 24 h after randomization.

These endpoints provide complementary renal and clinical assessments of the early treatment response. Total 24-h urinary sodium excretion quantifies the natriuretic response. Body weight change was selected as a pragmatic, non-urine-based clinical measure that is routinely available in hospital and ambulatory practice and does not require complete timed urine collection.

Secondary endpoints include changes in lower limb circumference at 5 and 10 cm above the ankle at 24 and 72 h; changes in clinical congestion score at 24 and 72 h; changes in IVC diameter at 3, 24, and 72 h; change in NT-proBNP at 72 h; change in CA-125 at 15 ± 3 days; cumulative furosemide-equivalent dose during the first 72 h after randomization; and 30-day safety.

Safety endpoints include adverse events related to compression; symptomatic hypotension; electrolyte disturbances at 72 h (hyponatraemia <135 mmol/L, hypernatraemia >150 mmol/L, hypokalaemia <3.5 mmol/L, and hyperkalaemia >5.0 mmol/L); worsening renal function (doubling of serum creatinine, estimated glomerular filtration rate decline ≥50% from baseline, renal replacement therapy); worsening heart failure events; and all-cause mortality. Worsening heart failure events are defined as hospitalization for heart failure or an urgent heart failure visit requiring parenteral diuretic therapy in an unscheduled ambulatory clinic or emergency department visit without an overnight stay. Recurrent episodes are counted separately when separated by at least 14 days of clinical stability; events occurring during the same hospitalization or continuous episode of care are counted as one event.

### Sample size

The sample size calculation is driven by the urinary sodium endpoint. Assuming a between-group difference of 20 mmol in 24-h urinary sodium excretion, a standard deviation of 30 mmol, two-sided α of .05, and 90% power, 96 patients are required. Allowing for approximately 10% incomplete endpoint ascertainment, the target sample size is 106 patients.

### Statistical analysis

Continuous variables will be summarized as mean (SD) or median (IQR), according to distribution. Categorical variables will be summarized as counts and percentages. Baseline comparisons will use Student's *t*-test or the Mann–Whitney *U* test for continuous variables and χ^2^ or Fisher’s exact test for categorical variables.

All analyses will follow the intention-to-treat principle. The co-primary endpoints will be compared between groups at the prespecified 24-h time point using mixed-effects regression models with centre included as a random effect. Secondary endpoints assessed repeatedly after randomization will be analysed using mixed-effects models with random effects for centre and patient, and treatment effects will be estimated across the relevant post-randomization visits. All models will be adjusted for a prespecified set of baseline covariates selected *a priori* on the basis of their established prognostic association with the natriuretic and diuretic response, independently of their observed between-group balance: log-transformed baseline NT-proBNP, baseline estimated glomerular filtration rate, baseline parenteral loop diuretic dose, prior heart failure hospitalization, and the Charlson Comorbidity Index. For secondary endpoints with a repeated-measures structure, treatment comparisons will be adjusted for multiplicity using the Šidák method. Given the modest sample size, the trial is designed as a proof-of-concept study; the prespecified covariate-adjusted model will constitute the primary analysis, with unadjusted estimates reported for transparency. Statistical significance will be defined as a two-sided *P* < .05. All analyses will be performed in Stata 19 by an independent statistician (MedStats Consulting, Reading, PA, USA)

### Trial status and baseline phenotype

**Figure 3:**
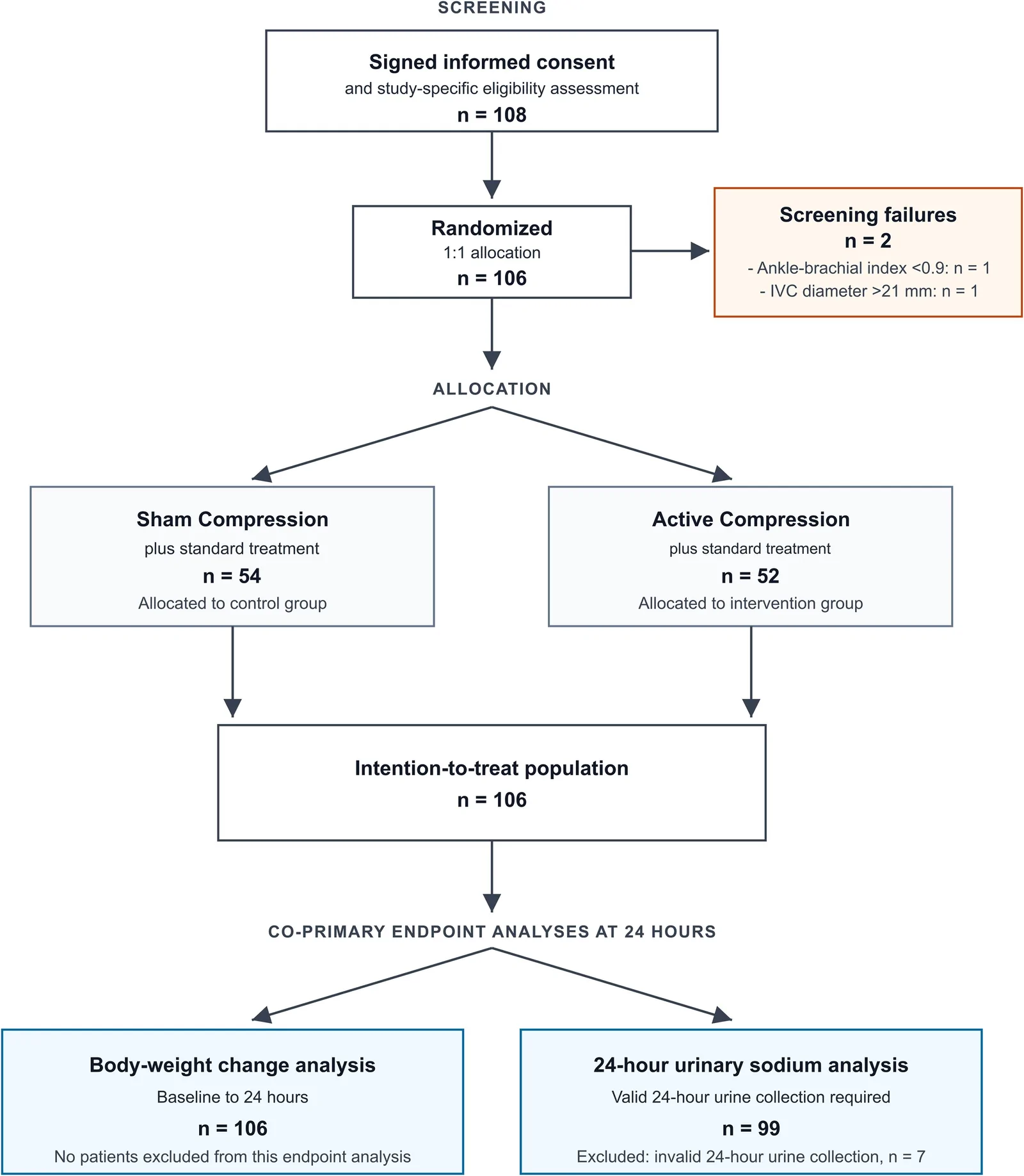
Participant flow. Of 108 patients who provided informed consent, 2 were screening failures (one ankle–brachial index <0.9; one inferior vena cava diameter >21 mm). A total of 106 patients were randomized: 54 to sham compression plus standard care and 52 to active compression plus standard care. All 106 patients were included in the 24-h body-weight analysis population; 99 patients had evaluable 24-h urinary sodium data

Recruitment is complete. Of 108 patients who provided written informed consent, 2 were screening failures: one because of an ankle–brachial index <0.9 and one because of an IVC diameter >21 mm. A total of 106 patients were randomized, 54 to sham compression plus standard care and 52 to active compression plus standard care. All 106 patients were included in the 24-h body weight analysis population; 99 patients had evaluable 24-h urinary sodium data after exclusion of invalid urine collections. Participant flow is shown in *Figure 3*.

Among the 106 randomized participants, 30 (28.3%) were enrolled during hospitalization and 76 (71.7%) in the ambulatory setting. In ambulatory participants, parenteral furosemide was initiated as part of routine clinical care on the day of screening and randomization, before written informed consent and independently of study participation; consent was obtained before any study-specific procedure. Among hospitalized participants, the median duration of parenteral treatment before randomization was 48 h (IQR, 0–48). The median parenteral-equivalent furosemide dose before randomization was 120 mg/24 h (IQR, 80–160).

The enrolled population reflected the intended phenotype (*Table 2*). Mean age was 82.1 years, 57 patients (53.8%) were men, and mean left ventricular ejection fraction was 53.5%; only 17 patients (16.0%) had left ventricular ejection fraction <40%. Comorbidity burden was high, with chronic kidney disease in 53 patients (50.0%), diabetes in 46 (43.4%), and atrial fibrillation or flutter in 70 (66.0%). Peripheral oedema was extensive: 102 patients (96.2%) had oedema reaching at least the knee. In contrast, markers of intravascular and pulmonary congestion were modest, with a mean IVC diameter of 18.0 mm, and jugular venous distension in 20 patients (18.9%). Median NT-proBNP was 4132 pg/ml, and median CA-125 was 54.5 U/mL.

**Table 2:** Baseline characteristics of the randomized population

	Sham compression (*n* = 54)	Active compression (*n* = 52)	Total (*n* = 106)
Demographic and clinical characteristics			
Age, years	81.8 ± 9.4	82.4 ± 8.9	82.1 ± 9.1
Male sex	29 (53.7%)	28 (53.8%)	57 (53.8%)
Ambulatory enrolment	37 (68.5%)	39 (75.0%)	76 (71.7%)
Prior HF hospitalization	20 (37.0%)	27 (51.9%)	47 (44.3%)
Atrial fibrillation or flutter	35 (64.8%)	35 (67.3%)	70 (66.0%)
Chronic kidney disease	24 (44.4%)	29 (55.8%)	53 (50.0%)
Diabetes mellitus	19 (35.2%)	27 (51.9%)	46 (43.4%)
eGFR, ml/min/1.73 m^2^	48.0 (35.5–62.1)	39.0 (28.7–53.8)	41.4 (32.5–59.6)
NT-proBNP, pg/ml	3535 (1589–6733)	5094 (2260–13 118)	4132 (1944–9956)
CA-125, U/ml	52.6 (19.8–166.8)	61.0 (24.5–125.3)	54.5 (23.2–135.7)
Physical examination			
Systolic blood pressure, mm Hg	127.1 ± 21.3	127.7 ± 21.8	127.4 ± 21.4
Heart rate, bpm	75 (67–86)	70 (62–75)	71 (65–83)
Peripheral oedema at or above knee level	52 (96.3%)	50 (96.2%)	102 (96.2%)
Jugular venous distension	11 (20.4%)	9 (17.3%)	20 (18.9%)
Pulmonary crackles	23 (42.6%)	24 (46.2%)	47 (44.3%)
Orthopnoea	17 (31.5%)	18 (34.6%)	35 (33.0%)
Clinical congestion score	3 (2–4)	3 (2–4)	3 (2–4)
Echocardiography			
Left ventricular ejection fraction, %	54.6 ± 12.2	52.5 ± 13.2	53.5 ± 12.7
LVEF <40%	8 (14.8%)	9 (17.3%)	17 (16.0%)
Inferior vena cava diameter, mm	18.0 ± 2.9	18.0 ± 2.7	18.0 ± 2.8
IVC collapsibility >50%	24 (46.2%)	24 (46.2%)	48 (46.2%)

Data are mean ± SD, median (interquartile range), or *n* (%).

CA-125, carbohydrate antigen 125; eGFR, estimated glomerular filtration rate; HF, heart failure; IVC, inferior vena cava; LVEF, left ventricular ejection fraction; NT-proBNP, N-terminal pro–B-type natriuretic peptide.

## Discussion

The main early treatment goal in patients presenting with ADHF is to achieve effective and safe decongestion. Loop diuretics remain the standard first-line treatment, but insufficient response is common and is associated with residual congestion and poor outcomes.^3,18^ COMPRESSION-HF evaluates whether adding lower limb compression to standard diuretic therapy improves early decongestion in a selected group of patients with predominant tissue congestion and no marked intravascular congestion.

The rationale for this study is based on the distinction between intravascular and tissue congestion. In clinical practice, some patients exhibit marked interstitial volume expansion despite no clear evidence of intravascular congestion. This pattern is more likely in clinical settings characterized by reduced plasma oncotic pressure, endothelial or glycocalyx dysfunction with increased capillary permeability, and impaired lymphatic drainage, including advanced age, chronic kidney disease, and obesity.^3,8^ In these patients, further intensification of diuretics may not be the best way to improve decongestion if the main limitation is insufficient fluid movement from the interstitial to the intravascular compartment.^19,20^

Recent studies using indocyanine green fluorescence lymphography have provided direct evidence that lower-extremity lymphatic flow is active but heterogeneous in acute heart failure with peripheral oedema. In an initial study, 13 of 20 patients (65%) had lymphatic flow beyond the ankle within 10 min at rest, whereas 7 (35%) had minimal or no detectable flow.^21^ In a subsequent cohort of 65 patients receiving protocolized intravenous furosemide, a longer distance of lymphatic flow was independently associated with a better diuretic response (odds ratio 1.48 per 10-cm increase, 95% CI, 1.08–2.03; *P* = .012).^22^ Although these observational findings do not establish the direction of effect or causality, they support the relevance of lymphatic transport to maintaining plasma refill during diuresis.

These considerations define the population targeted in COMPRESSION-HF. The IVC criterion identifies patients with less marked central venous congestion, not necessarily globally milder heart failure. The randomized cohort was elderly and had a substantial burden of chronic kidney disease, diabetes, atrial fibrillation, and extensive lower limb oedema, with elevated NT-proBNP concentrations despite modest IVC diameter and infrequent jugular venous distension. This profile is consistent with a clinically recognizable phenotype of persistent tissue congestion without marked central venous distension and defines the scope within which the results should be interpreted.

Because lower limb oedema is a frequent clinical finding in this phenotype, compression was selected as a simple mechanical intervention that can be applied by trained nursing staff. By increasing tissue pressure, compression may enhance venous and lymphatic return, improve plasma refill after loop diuretic administration, and make more sodium and water available for renal excretion. In the pilot study supporting COMPRESSION-HF, venous leg compression increased IVC diameter, particularly among patients with a baseline IVC diameter ≤21 mm, and this early refill response was accompanied by greater natriuresis and clinical decongestion.^17^ The present trial evaluates this approach in a randomized, sham-controlled setting.

The lymphatic system has also been evaluated as a therapeutic target in the DELTA-HF programme, which used an endovascular system to reduce pressure at the thoracic duct outflow and facilitate lymph return to the central circulation.^23^ In its multicentre, single-arm feasibility study, the system was successfully deployed, activated, and removed in all 40 treated patients, and a reduced-pressure thoracic duct zone was achieved in 39 (97.5%). Improvements across several congestion measures provided an early clinical signal, but the absence of a control group, the small sample size, and two procedure-related serious adverse events, including one death, preclude conclusions regarding efficacy.^24^ These findings support further randomized evaluation of lymphatic-targeted decongestion and provide complementary rationale for studying a non-invasive tissue-directed strategy in COMPRESSION-HF. Because lymphatic flow was not directly measured in COMPRESSION-HF, enhanced lymphatic drainage remains a plausible rather than demonstrated mechanism of lower limb compression.

Accordingly, the co-primary endpoints were selected to provide complementary renal and clinical assessments. Total 24-h urinary sodium excretion provides an objective measure of the natriuretic response and is increasingly used to evaluate decongestive strategies in acute heart failure.^25,26^ Body weight change was selected as a pragmatic clinical endpoint because it is routinely available in hospital and ambulatory practice and does not depend on complete timed urine collection. It is not a one-to-one measure of diuresis or net fluid balance; substantial discordance between recorded fluid balance and measured weight change has been demonstrated during treatment for ADHF.^27^ Selecting urine volume as the other co-primary endpoint would have made both co-primary endpoints dependent on the same 24-h urine collection. Serial IVC diameter, lower limb circumference, biomarkers, renal function, diuretic exposure, and safety therefore provide complementary information on intravascular refill, local tissue response, and clinical tolerability.

If the trial is positive, lower limb compression would be a simple adjunct to diuretic therapy in a well-defined group of patients. Its implementation would mainly require confirmation of the target phenotype, predominant tissue congestion without marked intravascular congestion, and exclusion of standard contraindications to compression, particularly peripheral arterial disease.

The main strengths of COMPRESSION-HF are its randomized, double-blind, sham-controlled design, selection of a clinically relevant congestion phenotype, and use of endpoints that assess both natriuretic and clinical responses. The trial also retains a pragmatic structure: the target phenotype can be identified at the bedside using focused IVC assessment, the intervention uses a commercially available system, and application can be performed by trained nursing staff.

Several limitations should be recognized. Interface pressure was not measured directly; therefore, interindividual variation in achieved pressure and changes between renewals cannot be excluded. Changes in lower limb circumference may reflect both local mechanical displacement of interstitial fluid and systemic fluid removal. Because measurements were obtained during scheduled bandage renewal, this endpoint cannot distinguish transient tissue compression from sustained tissue decongestion and should be interpreted with the natriuretic, body weight, clinical congestion, and intravascular refill endpoints. Finally, the selected phenotype and exclusion of patients with marked IVC distension or peripheral arterial disease limit extrapolation to broader populations with acute heart failure.

In conclusion, COMPRESSION-HF will determine whether lower limb compression therapy improves early natriuresis and weight loss in patients with decompensated heart failure, predominant peripheral tissue congestion, and IVC diameter ≤21 mm. The study will also provide prospective information on the relationship between tissue congestion, intravascular refill, urinary sodium excretion, and clinical decongestion during treatment for ADHF.

## Declarations

### Data Availability

No data were generated or analysed for this manuscript.

### Pre-registered Clinical Trial Number

ClinicalTrials.gov NCT06418932
